# Structural and Sensory Impacts of Black Garlic Particle–Based Fat Mimetics in Low‐Fat Mayonnaise

**DOI:** 10.1155/ijfo/7746086

**Published:** 2026-07-11

**Authors:** Avido Yuliestyan, Yulius Deddy Hermawan, Lisendra Marbelia, Ningrum Arum Sari, Gloria Bagas Puspakirana, Riyan Hidayat

**Affiliations:** ^1^ Department of Chemical Engineering, Faculty of Industrial Engineering, Universitas Pembangunan Nasional “Veteran” Yogyakarta, Yogyakarta, Indonesia, upnyk.ac.id; ^2^ Department of Chemical Engineering, Faculty of Engineering, Universitas Gadjah Mada, Yogyakarta, Indonesia, ugm.ac.id

**Keywords:** black garlic particles, emulsion rheology, fat mimetic, low-fat mayonnaise, protein–polysaccharide interaction

## Abstract

Reducing fat content in mayonnaise often compromises structural integrity and sensory quality. This study investigated black garlic particles (BGPs) as a particulate fat mimetic in low‐fat mayonnaise formulated with a whey protein concentrate (WPC)–high‐methoxyl pectin (HMP) matrix. Mayonnaise samples containing 4–16 g BGP were evaluated for physicochemical properties, microstructure, rheological behavior, short‐term stability, and sensory acceptance. All formulations exhibited non‐Newtonian shear‐thinning behavior (n < 1). Among the tested samples, BGP‐12 showed rheological properties closest to conventional mayonnaise, with a consistency index of approximately 3.87 Pa·s^n^ and elastic‐dominant viscoelastic behavior (G ^′^ > G ^″^). FTIR and microstructural analyses suggested that BGP reinforced the protein–polysaccharide matrix through noncovalent interactions, contributing to emulsion structuring and stability. Short‐term storage (7 days) revealed minimal structural changes and a slight increase in elastic behavior over time. Sensory evaluation by 57 untrained panelists showed high acceptance for viscosity (4.11 ± 0.70; 84.2%) and texture (3.58 ± 1.00; 64.9%). The optimized formulation achieved approximately 75% fat reduction and an estimated 45%–55% reduction in caloric content while maintaining key functional and sensory attributes. These findings demonstrate the potential of BGP as an effective particulate fat mimetic by reinforcing the protein–polysaccharide network through filler–matrix interactions, for developing structurally stable and sensory‐acceptable low‐fat mayonnaise.

## 1. Introduction

The increasing consumption of high‐fat foods has become a major global health concern. According to the World Health Organization, cardiovascular diseases account for approximately 19.8 million deaths worldwide, with unhealthy dietary patterns being a major contributing factor. Diets characterized by high intake of foods rich in fat, sugar, and salt (HFSS), as well as ultraprocessed foods (UPFs), are strongly associated with obesity and chronic noncommunicable diseases [1, 2]. Consequently, there is growing interest in developing healthier food products, including low‐fat alternatives, to improve public health outcomes [3, 4].

Fat plays a crucial role in food systems by contributing to texture, appearance, mouthfeel, and flavor. In emulsified products such as mayonnaise, fat not only enhances sensory attributes but also governs emulsion structure by regulating the distribution of oil and water phases. Reducing fat content often leads to undesirable changes in physicochemical and sensory properties, including reduced viscosity, poor mouthfeel, and emulsion instability [5]. Therefore, successful fat reduction strategies require fat‐mimetic ingredients capable of reproducing the functional and sensory roles of fat without increasing lipid content.

Food emulsions are thermodynamically unstable systems composed of immiscible oil and water phases that tend to separate over time unless stabilized by emulsifiers [4, 6]. Conventional emulsions are commonly stabilized using surfactants or amphiphilic polymers; however, such systems may suffer from limited long‐term stability and raise environmental and safety concerns due to poor biodegradability or potential irritation effects [7, 8]. In mayonnaise production, egg yolk is traditionally used as an emulsifier, but its use results in high fat content and presents food safety risks, including *Salmonella* contamination and allergenicity concerns [9]. These limitations highlight the need for natural, sustainable, and clean‐label emulsifier alternatives.

Fat mimetics derived from proteins and polysaccharides have gained increasing attention due to their ability to mimic fat‐like texture and mouthfeel while improving emulsion stability. Polysaccharides such as pectin can form physically structured systems through intermolecular interactions, increasing viscosity and contributing to physical stability [10]. In addition, particulate‐based systems can act as stabilizers in Pickering‐type emulsions, providing enhanced resistance to coalescence and improved stability [7, 11]. Despite increasing interest in fat mimetics based on protein–polysaccharide systems, the mechanistic role such systems play in enhancing viscoelastic properties remains poorly understood. In particular, the relationship between particle‐induced microstructural changes and elastic network formation has not been clearly established. Furthermore, it remains unclear whether such rheological improvements translate into enhanced sensory perception and consumer acceptance.

Black garlic, produced through controlled thermal fermentation of fresh garlic, has emerged as a functional ingredient of interest. It is rich in polysaccharides, phenolic compounds, and Maillard reaction products such as melanoidins, which contribute to its high antioxidant activity, water‐holding capacity, and potential structural functionality [12, 13]. These characteristics suggest that black garlic particles (BGPs) may serve not only as bioactive ingredients but also as multifunctional fat mimetics by enhancing viscosity, stabilizing emulsions, and modifying texture through particle–matrix interactions. Despite these promising properties, the application of BGP as a fat mimetic in emulsified systems such as mayonnaise remains largely unexplored.

Based on this background, the present study investigates the use of BGPs as a fat‐mimetic component in low‐fat mayonnaise, with a primary focus on explaining its role in modifying the microstructure of protein–polysaccharide systems. Furthermore, the effects of BGP incorporation on rheological behavior, microstructure, physical appearance, sensory acceptance, and nutritional profile are systematically evaluated to establish the relationship between structural functionality and product acceptability.

## 2. Materials and Methods

### 2.1. Materials and Formulations

Whey protein concentrate (WPC; 85% protein) was obtained from a local supplier and used as the protein‐based structuring agent. High‐methoxyl pectin (HMP) was used as the polysaccharide component in the fat‐mimetic system, whereas low‐methoxyl pectin (LMP) was used solely as a reference material for Fourier transform infrared (FTIR) spectroscopy analysis. Those HMP and LMP were obtained from CP Kelco, Lille Skensved, Denmark. Vinegar purchased from a local market served as the acidifying agent in the mayonnaise formulation, whereas sugar and salt, also sourced locally, were added for flavor adjustment. Soybean oil was used as the lipid phase in both conventional and low‐fat mayonnaise formulations. All materials were used as received without further purification.

### 2.2. Preparation of BGPs

Fresh garlic obtained from a local market was subjected to thermal fermentation to produce black garlic. The fermentation process was carried out at 65°C for 20 days in a temperature‐controlled chamber, following conditions adapted from Zhao et al. [13] to promote desirable physicochemical transformations. The relative humidity during fermentation was maintained at approximately 80%, in accordance with typical black garlic–processing conditions reported in the literature. The resulting black garlic was then processed into particles, and the particle size was standardized by sieving through a 140‐mesh sieve (~100 *μ*m). The particle size distribution was considered relatively uniform based on the sieving method and beyond the scope of the present study and should be considered in future investigations.

### 2.3. Preparation of Mayonnaise

**Figure 1:**
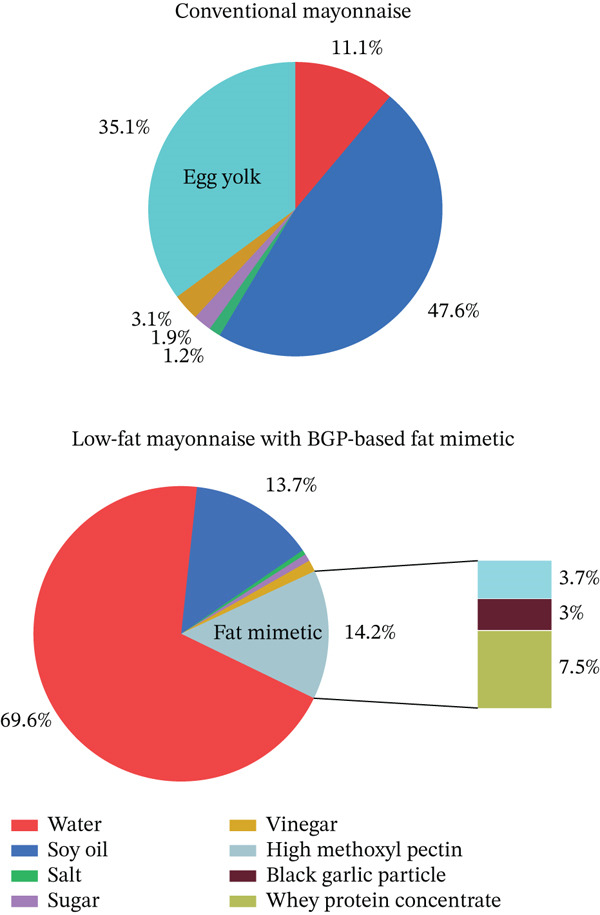
Comparison of ingredient composition between conventional mayonnaise and low‐fat mayonnaise formulated with a BGP‐based fat mimetic.

Fat mimetics were prepared prior to mayonnaise production, as illustrated in Figure 1. The fat‐mimetic base was formulated by dispersing 30 g of WPC and 15 g of HMP in 280 mL of distilled water under continuous mixing until a homogeneous dispersion was obtained [10].

For BGP‐containing mayonnaise formulations, 60 mL of soybean oil and the designated amount of BGPs (4, 8, 12, or 16 g) were added to the fat‐mimetic base and homogenized at 1500 rpm for 10 min. The BGP concentration range (4–16 g) was selected based on Du et al. [10], who reported stable whey protein–HMP complexes at pectin concentrations above 1.5%. Preliminary trials were first conducted to obtain a low‐fat mayonnaise with a viscosity comparable with commercial mayonnaise (~12.2 Pa·s at low shear rate). Based on these results, BGP concentrations spanning below and above the reference level were chosen to evaluate their effects on formulation performance. The mixture was then combined with sugar, salt, and vinegar and further mixed to obtain a stable emulsion. Conventional mayonnaise was prepared as a control formulation. Egg yolk (57 g) was first whisked until a thick and frothy consistency was achieved, followed by the addition of sugar, salt, and vinegar. Soybean oil (84 mL) and water (18 mL) were then gradually incorporated while homogenizing the mixture using a hand blender until a stable emulsion was formed [14]. The formulation was defined based on fixed quantities of each component, and the corresponding proportions are implicitly represented by the given masses and volumes, ensuring reproducibility of the system.

### 2.4. Tests and Measurements

#### 2.4.1. FTIR Analysis

FTIR analysis was conducted to determine the chemical composition and identify functional groups associated with polysaccharides and proteins in BGPs and pectin. Spectra were recorded using a Thermo Scientific Nicolet iS10 FTIR spectrometer over a wavenumber range of 4000–400 cm^−1^, with a resolution of 4 cm^−1^ and 50 scans per sample. Spectra were collected in absorbance mode and analyzed to assess characteristic functional group vibrations.

#### 2.4.2. Physical Appearance Analysis

The physical appearance of mayonnaise samples was evaluated qualitatively in terms of color, homogeneity, and presence of particles. This preliminary assessment was used to identify macroscopic differences among formulations prior to further microstructural, rheological, and sensory analyses.

#### 2.4.3. Microstructure Observation

Optical micrographs were obtained to examine the microstructure of the mayonnaise samples. An Optilab Iris 4 trinocular microscope was used at magnifications of 4× and 10×. Samples were gently placed on microscope slides without dilution to avoid structural disruption. Microstructural observations focused on oil droplet distribution and matrix homogeneity. Micrographs were analyzed qualitatively to compare differences among formulations. Although quantitative droplet size distribution analysis was not performed, the microstructural observations were interpreted in conjunction with rheological measurements to evaluate emulsion stability.

#### 2.4.4. Viscosity and Rheological Measurements

Initial viscosity measurements were performed using a Brookfield DV2T viscometer equipped with spindle LV‐64 over a rotational speed range of 5–150 rpm. Measurements were conducted for 2 min at each speed, with data recorded at 30 s intervals, to evaluate consistency relative to typical commercial mayonnaise.

Shear flow rheological measurements were carried out using a Modular Compact Rheometer (Anton Paar MCR 102) fitted with a 25‐mm plate geometry and a gap of 1 mm at 25°C. Apparent viscosity was measured over a shear rate range of 0.1–500 s^−1^ to assess shear‐thinning behavior.

Dynamic viscoelastic properties, including storage modulus (G ^′^), loss modulus (G ^″^), and loss tangent (tan *δ*), were determined using oscillatory frequency sweep tests conducted from 0.1 to 10 Hz at a constant strain of 0.1%, which was within the linear viscoelastic region.

All measurements were performed in quadruplicate. Data processing and visualization were conducted using OriginPro 2023.

#### 2.4.5. Sensory Evaluation

A hedonic‐type sensory evaluation was conducted to assess consumer acceptance of individual sensory attributes of the selected low‐fat mayonnaise formulation containing fat mimetics. The evaluation was performed using a 5‐point hedonic scale and involved 57 untrained panelists aged between 17 and 53 years. Panelists evaluated aroma, taste, texture, viscosity, and color, and overall liking to identify sensory drivers and potential limitations of the formulation.

#### 2.4.6. Statistical Analysis

Statistical analysis was performed using one‐way analysis of variance (ANOVA) followed by Tukey′s post hoc test to evaluate the effect of BGP concentration, with significance determined at *p* < 0.05.

## 3. Results and Discussion

### 3.1. FTIR Analysis

BGPs were hypothesized to exhibit molecular features comparable with those of LMP, particularly with respect to their polysaccharide‐rich composition and abundance of hydroxyl and carboxyl functional groups. Although HMP was employed in the formulation of the fat‐mimetic system, LMP was selected as a reference material for FTIR analysis due to its well‐defined spectral characteristics and structural similarity to plant‐derived polysaccharides. The comparative FTIR analysis was therefore conducted to evaluate whether BGP possess functional group signatures consistent with pectin‐like biopolymers that could contribute to physical stabilization through noncovalent interactions.

**Figure 2:**
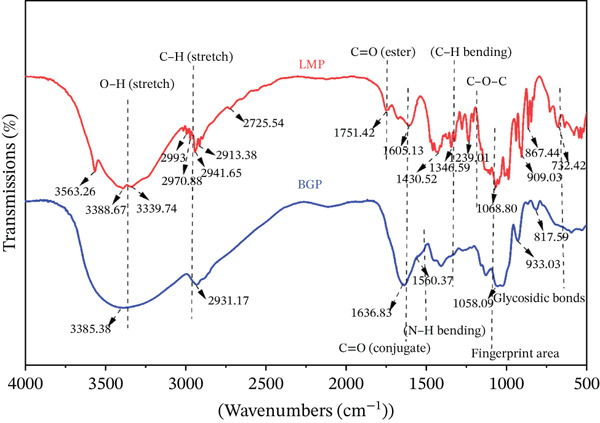
FTIR spectra of black garlic particle and low‐methoxyl pectin.

The FTIR spectra of LMP and BGPs (Figure 2) reveal distinct yet complementary molecular features that underpin their roles in the fat‐mimetic emulsion system. Both materials exhibit a broad O–H stretching band centered around 3400 cm^−1^, indicative of abundant hydroxyl groups and extensive hydrogen bonding, which are characteristic of polysaccharide‐ and protein‐containing biopolymers [15]. These hydroxyl groups facilitate intermolecular interactions within the continuous phase, contributing to viscosity enhancement and physical stabilization of the emulsion matrix.

In the LMP spectrum, two characteristic absorptions are observed in the carbonyl region. A weak ester C=O stretching band appears at approximately 1730–1750 cm^−1^, whereas a more intense asymmetric COO^−^ stretching band is present at around 1600–1630 cm^−1^. The predominance of the COO^−^ band reflects the low degree of esterification of the pectin and a high density of free carboxyl groups, as commonly determined by FTIR analysis [16]. These negatively charged carboxyl groups promote electrostatic repulsion between oil droplets and contribute to continuous‐phase structuring, supporting the function of LMP as both a polymeric thickener and a stabilizing agent in emulsified systems.

In contrast, the FTIR spectrum of BGP exhibits distinct Amide I and Amide II bands at approximately 1650 and 1550 cm^−1^, respectively, confirming the presence of proteinaceous components and peptide backbone structures [17]. The fingerprint region below 1200 cm^−1^ shows complex overlapping bands arising from carbohydrate structures and thermally generated compounds formed during black garlic fermentation. These spectral features are consistent with the formation of Maillard reaction products, including melanoidins, which have been widely reported in black garlic [12, 18]. Although FTIR does not allow direct identification of individual Maillard products, the increased spectral complexity suggests a heterogeneous particulate composition capable of participating in multiple noncovalent interactions.

Importantly, no new absorption bands or significant peak shifts were observed in the combined system, indicating that emulsion stabilization is dominated by physical interactions rather than covalent bond formation. The coexistence of hydroxyl, carboxyl, and amide functional groups supports a stabilization mechanism driven by hydrogen bonding, electrostatic interactions, and steric effects between protein and polysaccharide components. These reversible interactions are consistent with the rheological behavior observed for the optimized formulation, which exhibits enhanced elastic response at rest while remaining shear thinning under applied deformation. These molecular features further support the ability of BGP to interact with protein polysaccharide networks in the continuous phase, thereby contributing to increased viscosity and improved emulsion stability observed at the macroscopic level. Overall, the FTIR results support the role of BGPs as multifunctional components within the fat‐mimetic matrix. It should be noted that FTIR provides only indirect evidence of protein–polysaccharide interactions through the presence of relevant functional groups and spectral features. Therefore, the proposed stabilization mechanism is inferred from the combined FTIR, rheological, and microstructural observations.

### 3.2. Physical Appearance

**Figure 3:**
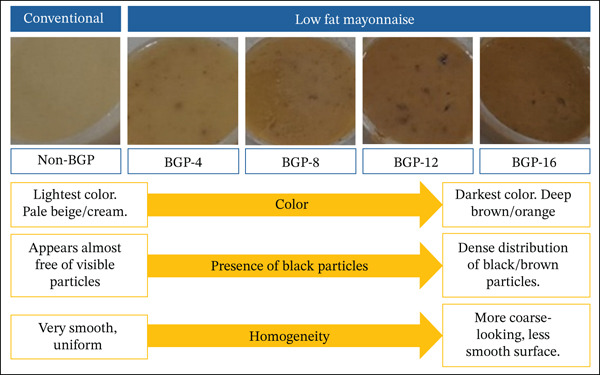
Visual appearance of low‐fat mayonnaise with black garlic–based fat mimetics.

Figure 3 illustrates the visual appearance of conventional mayonnaise and low‐fat mayonnaise samples formulated with varying concentrations of BGP. A progressive darkening in color is observed with increasing BGP content, with the BGP‐16 sample exhibiting the darkest hue, characterized by a deep brown–orange appearance. In contrast, the conventional mayonnaise without BGP (non‐BGP) displays the lightest color, appearing pale beige/cream. Beyond color variation, the presence of visible dark particles becomes more pronounced as the BGP concentration increases, reflecting the dispersion of black garlic solids within the emulsion matrix. The conventional mayonnaise (non‐BGP sample) appears nearly free of visible particles and exhibits a smoother and more homogeneous surface. In comparison, the low‐fat mayonnaise is stabilized using WPC and HMP as fat mimetics. At higher BGP levels, the mayonnaise exhibits a comparatively coarser visual heterogeneity and reduced surface smoothness.

However, traditional mayonnaise is typically associated with a light, creamy color indicative of freshness and quality. The progressive darkening in BGP‐containing samples may therefore affect consumer perception, particularly at higher concentrations, and will be further evaluated through sensory analysis in the subsequent section.

### 3.3. Microstructure Visualization

**Figure 4:**
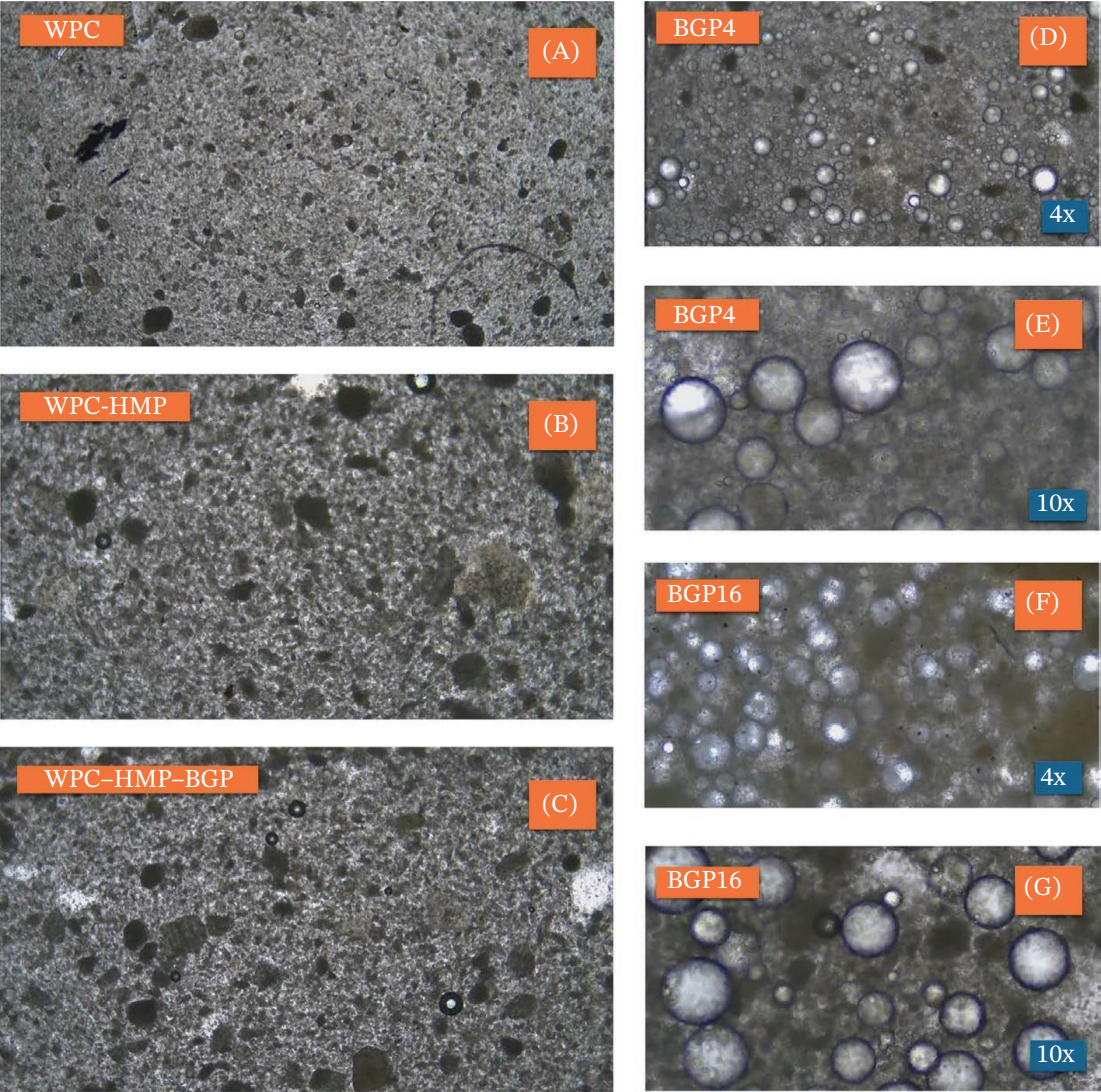
Representative optical micrographs of fat‐mimetic systems and mayonnaise formulations. (A) WPC, (B) WPC–HMP, (C) WPC–HMP–BGP, (D–E) BGP‐4 mayonnaise, and (F–G) BGP‐16 mayonnaise at different magnifications.

Figure 4A–C presents the optical microstructures of the fat‐mimetic systems composed of WPC, WPC–HMP, and WPC–HMP–BGPs. All systems display a continuous matrix without evidence of macroscopic phase separation, indicating good compatibility among the biopolymer components. The WPC‐only system exhibits the most homogeneous appearance, characterized by a relatively smooth background and uniformly distributed dispersed domains. This homogeneity reflects the ability of whey proteins to form a coherent continuous phase through hydration and intermolecular interactions [19].

The incorporation of HMP into the WPC matrix leads to increased microstructural heterogeneity, as evidenced by the appearance of larger and more irregular dispersed domains and a broader size distribution. Such behavior is characteristic of mixed protein–polysaccharide systems, in which partial segregation and competition for spatial distribution can occur without leading to visible phase separation [20] Despite the increased heterogeneity, the overall continuity of the matrix is maintained, suggesting that protein–polysaccharide interactions contribute to bulk phase structuring rather than network formation.

The addition of BGP to the WPC–HMP system does not introduce new or distinct microstructural features at the magnification employed. The microstructure of the WPC–HMP–BGP system appears visually comparable with that of the WPC–HMP formulation, indicating that BGPs are dispersed within the existing protein–polysaccharide matrix rather than forming an interconnected particle network. This observation suggests that BGP may contribute as filler‐like components that modulate matrix heterogeneity within an already continuous protein–polysaccharide matrix without fundamentally altering the underlying structural organization.

Figure 4D–G shows representative optical micrographs of mayonnaise emulsions containing low (BGP‐4) and high (BGP‐16) concentrations of BGPs. The BGP‐4 formulation exhibits relatively small and more uniformly distributed oil droplets, consistent with a visually homogeneous emulsion structure and effective stabilization by the continuous phase. In contrast, the BGP‐16 formulation displays a broader droplet size distribution (as observed qualitatively due to optical resolution limitation) and localized droplet clustering, indicating increased structural heterogeneity at higher particle loadings. Such droplet association is commonly observed in protein‐rich emulsions when excessive dispersed material limits interfacial flexibility and promotes flocculation rather than improved droplet dispersion [21, 22]. This behavior may lead to the formation of a more rigid structure, enhancing elasticity.

In both formulations, the absence of large‐scale droplet coalescence or compact particle aggregation suggests that the emulsions remain physically stable at the microscopic level. The diffuse regions observed around oil droplets are consistent with the presence of protein and polysaccharide components in the continuous phase, supporting a stabilization mechanism dominated by hydration forming a reinforced layer that leads to increased viscosity and steric hindrance [23]. Overall, the microstructural observations indicate that emulsion organization is governed primarily by the balance between protein–polysaccharide interactions and particulate loading, with intermediate BGP concentrations providing a more uniform microstructure that aligns with the observed rheological and sensory performance in the subsequent section.

### 3.4. Flowability, Viscoelastic Behavior, and Short‐Term Stability of Low‐Fat Mayonnaise

**Figure 5:**
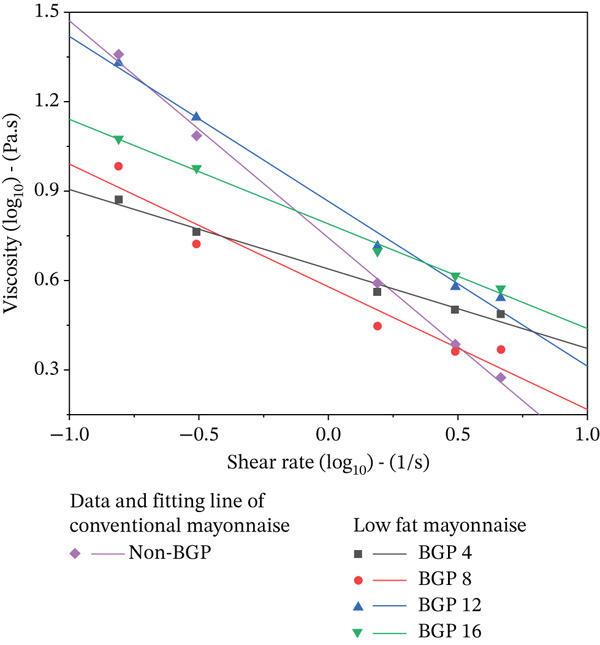
Flow behavior of conventional and low‐fat mayonnaise as a function of shear rate. Log–log plots of apparent viscosity versus shear rate show pronounced shear‐thinning behavior for all samples.

The flow behavior of all mayonnaise formulations (Figure 5) indicates that the samples were well described by the power‐law model (Equation 1), with high coefficients of determination (R^2^ > 0.94), confirming non‐Newtonian behavior.

(1) η=K.γ.n−1

All formulations exhibited shear‐thinning behavior (n < 1), which is a characteristic of mayonnaise systems and concentrated emulsions, where apparent viscosity decreases with increasing shear rate due to progressive disruption and alignment of the internal structure under flow [24, 25]. This behavior is technologically relevant, as it allows mayonnaise to remain thick and stable at rest while flowing readily during processing, spreading, or dispensing.

To quantitatively assess the influence of BGP concentration on viscosity, one‐way ANOVA was performed (Table 1). The results showed that there was a statistically significant effect across all shear rates (*p* < 0.05) with *F* values increasing progressively from 70.23 at SR1 to 644.99 at SR5. This trend indicates that the variability between formulations was substantially greater than the variability within replicates, confirming the strong influence of BGP incorporation on the system structure.

**Table 1:** Statistical significance results (one‐way ANOVA) assessing the effect of BGP concentration on viscosity at different shear rates.

Shear rate	*F*value	*p*value
SR1 (0.15 s^−1^)	70.23	1.57 × 10^−9^
SR2 (0.31^−1^)	166.30	3.14 × 10^−12^
SR3 (1.55^−1^)	149.16	6.95 × 10^−12^
SR4 (3.09^−1^)	286.95	5.67 × 10^−14^
SR5 (4.64^−1^)	644.99	1.39 × 10^−16^

Tukey′s post hoc analysis (Table 2) further showed that no single BGP formulation consistently reproduced the rheological behavior of conventional mayonnaise across all shear rates. At low shear (SR1), BGP‐12 exhibited no significant difference compared with the conventional sample (*p* > 0.05), indicating comparable initial viscosity. However, this similarity was not maintained at higher shear rates, where all BGP formulations differed significantly (*p* < 0.05), highlighting distinct shear‐dependent structural responses.

**Table 2:** Effect of black garlic particle (BGP) concentration on apparent viscosity at different shear rates (SR1–SR5) with Tukey′s post hoc grouping.

Sample	Mean viscosity (mPas) @				
	SR1	SR2	SR3	SR4	SR5
Conventional	22,920 (A)	12,225 (B)	3906 (B)	2431.5 (D)	1877 (E)
BGP‐4	7440 (C)	5805 (D)	3639 (B)	3174 (C)	3067 (C)
BGP‐8	9630 (BC)	5280 (D)	2799 (C)	2296.5 (D)	2334 (D)
BGP‐12	21,390 (A)	14,085 (A)	5202 (A)	3787.5 (B)	3481 (B)
BGP‐16	11,910 (B)	9510 (C)	4980 (A)	4135.5 (A)	3741 (A)

Further insight into this behavior is provided by the power‐law parameters (consistency index, K; flow behavior index, n; and coefficient of determination, R^2^) summarized in Table 3. The conventional mayonnaise (non‐BGP) exhibited the highest consistency index (K = 4.84 Pa · s^n^) and the lowest flow behavior index (n = 0.28), indicating a highly structured system with strong resistance to flow at low shear rates and pronounced shear sensitivity. This rheological response reflects the high oil volume fraction and the presence of egg yolk–derived emulsifiers, which promote dense droplet packing and strong interdroplet interactions, resulting in a rigid structure at rest that readily yields under shear.

**Table 3:** Consistency index, power law index, and R^2^.

Mayonnaise sample	Consistency index—K (Pa.*s* ^*n*^)	Power law index—*n*	*R* ^2^
Non‐BGP (conventional)	4.8429 ± 0.12	0.28 ± 0.01	0.9978
BGP‐4	3.6384 ± 0.08	0.74 ± 0.02	0.9844
BGP‐8	3.5788 ± 0.06	0.59 ± 0.03	0.9434
BGP‐12	3.8661 ± 0.10	0.45 ± 0.02	0.9910
BGP‐16	3.7961 ± 0.09	0.64 ± 0.02	0.9926

In contrast, all low‐fat formulations containing BGPs showed lower K values (3.58–3.87 Pa·s^n^), corresponding to reduced overall consistency and increased flowability due to the lower oil content and partial replacement of the fat phase by a protein–polysaccharide–based matrix. Among these formulations, BGP‐4 exhibited the highest n value (0.74), indicating weaker shear‐thinning behavior and a more freely flowing system. This response suggests insufficient structural reinforcement at low BGP levels, where the fat‐mimetic matrix provides limited resistance to deformation [26].

Increasing the BGP content to 8 and 12 g resulted in lower n values accompanied by moderately higher K values, indicating partial recovery of the internal structure and enhanced resistance to flow at low shear rates. The higher k indexes indicate the higher effective volume fraction occupied by BGPs leading to enhanced viscosity of the continuous phase, which reduces droplet mobility and limits coalescence. This trend suggests that increased particulate loading contributes to bulk phase structuring through filler effects and physical interactions with the protein–polysaccharide matrix, including hydrogen bonding and steric hindrance, thereby reinforcing the emulsion structure and yielding a flow response that more closely resembles that of conventional mayonnaise. At the highest BGP level (BGP‐16), the increase in n indicates reduced shear sensitivity, which may be attributed to increased structural heterogeneity and localized droplet association rather than further strengthening of the continuous network [25]. Similar trends have been reported in emulsions stabilized with fiber‐based fat mimetics, such as cellulose and polysaccharide systems [26].

Based on the combined analysis of consistency index (K) and flow behavior index (n), the BGP‐12 formulation exhibited the closest rheological resemblance to conventional mayonnaise, as indicated by the lowest deviation in consistency index (≈20.2%) and the closest flow behavior index (n = 0.45) relative to the control (n = 0.28). This formulation achieves a balance between sufficient structural integrity at rest and acceptable flowability under shear, supporting its selection as the most representative low‐fat mayonnaise system for further evaluation.

**Figure 6:**
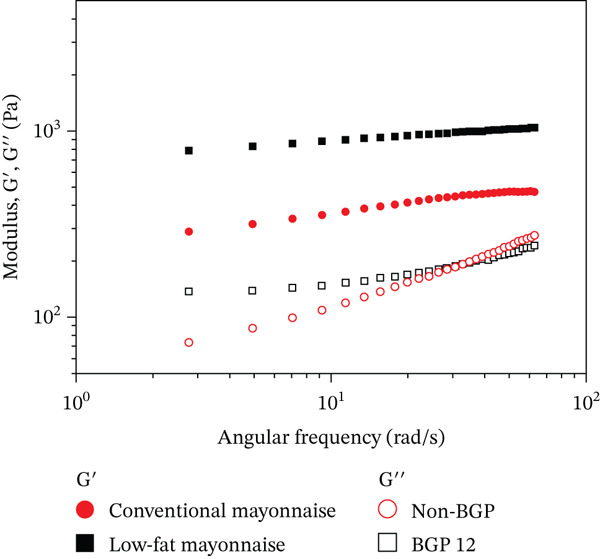
Viscoelastic properties of storage modulus (G ^′^) and loss modulus (G ^″^) with angular frequency of conventional mayonnaise and low‐fat mayonnaise.

The viscoelastic behavior of conventional mayonnaise and the selected low‐fat formulation (BGP‐12) is presented in Figure 6. For both samples, the storage modulus (G ^′^) consistently exceeds the loss modulus (G ^″^) across the entire frequency range, indicating predominantly elastic, solid‐like behavior under small‐amplitude oscillatory shear. This response is a characteristic of mayonnaise‐type emulsions and reflects the presence of a structured internal organization within the continuous phase capable of storing deformation energy rather than dissipating it as viscous flow [27]. Such elastic dominance is a key requirement for mayonnaise stability, as it limits droplet mobility and contributes to resistance against gravitational separation during storage.

Notably, the BGP‐12 formulation exhibits higher G ^′^ and G ^″^ values than the conventional mayonnaise throughout the tested frequency range, indicating a greater resistance to small deformations and a more elastic structure at rest, despite its substantially lower fat content. This increase in viscoelastic moduli suggests that the internal structure of BGP‐12 is reinforced by the fat‐mimetic matrix, compensating for the reduced oil volume fraction. The higher G ^′^ values reflect enhanced elastic structuring of the continuous phase, whereas the concomitant increase in G ^″^ indicates greater energy dissipation during deformation, consistent with a more densely structured yet still deformable system.

The enhanced elasticity of BGP‐12 can be attributed to the combined presence of WPC and pectin, which are known to promote elastic structuring in reduced fat emulsions through intermolecular associations and continuous‐phase reinforcement [10]. These biopolymers form a physically associated matrix through reversible interactions that restrict droplet movement and increase resistance to deformation. This interpretation is further supported by FTIR and microstructural observations, which indicate the predominance of reversible physical interactions, including hydrogen bonding and electrostatic interactions between protein and polysaccharide components. Such interactions give rise to a weak, elastic, biopolymer‐mediated structure, which remains intact under small deformations while progressively yielding under applied shear [10].

Overall, the combined flow and viscoelastic results demonstrate that the BGP‐12 formulation achieves a favorable balance between elastic solidity at rest and flowability under shear. This balance is essential for maintaining structural stability during storage while ensuring acceptable handling and sensory performance during use [14], thereby supporting the selection of BGP‐12 as the most representative low‐fat mayonnaise formulation relative to the conventional system.

**Figure 7:**
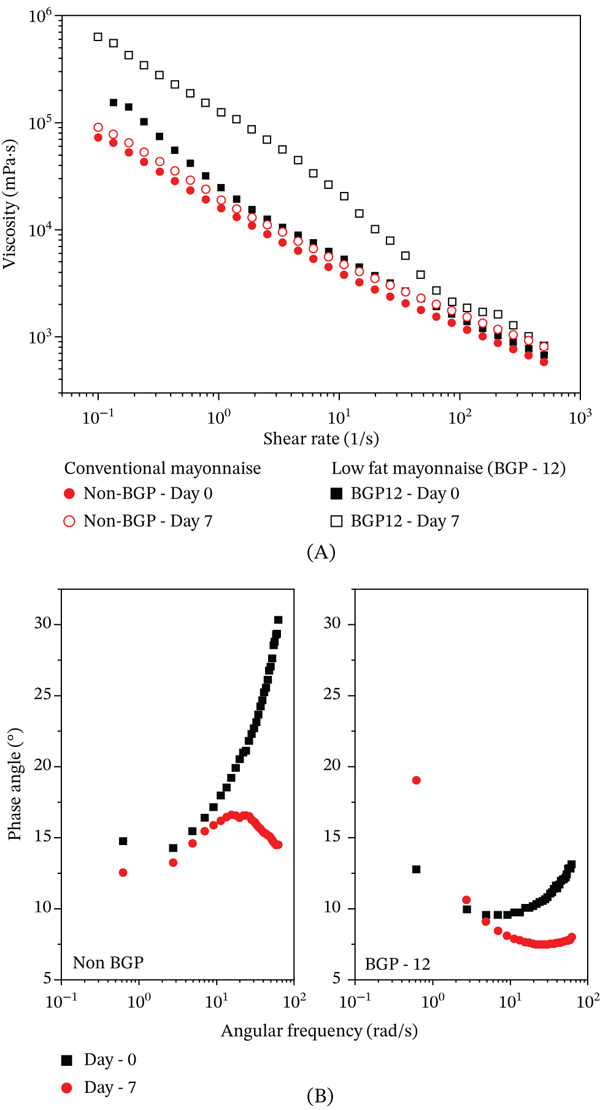
Rheological stability of conventional and low‐fat mayonnaise during 7 days of storage. (A) Apparent viscosity as a function of shear rate and (B) phase angle as a function of angular frequency for conventional mayonnaise (non‐BGP) and low‐fat mayonnaise containing 12% BGP.

Figure 7 compares the rheological evolution of conventional mayonnaise and the selected low‐fat formulation (BGP‐12) at Day 0 and after 7 days of storage. Short‐term stability (7 days) was selected to evaluate early structural evolution. Both systems retain pronounced shear‐thinning behavior after storage, indicating that their non‐Newtonian flow characteristics are preserved over time. This persistence of shear‐thinning behavior suggests that the fundamental internal structure of the emulsions remains intact, with no evidence of large‐scale coalescence or catastrophic structural breakdown during storage [28].

Only moderate changes in apparent viscosity are observed, predominantly at low shear rates, where the emulsion structure is most sensitive to weak interactions and long‐range organization. Such changes are indicative of limited structural rearrangement rather than irreversible degradation of the emulsion structure. At higher shear rates, the viscosity profiles of both formulations remain largely unchanged, reflecting the dominance of shear‐induced alignment and disruption of the internal structure during flow [29]. This behavior is consistent with physically stabilized emulsions for overall flow response.

The phase angle (*δ*) results further reveal a shift toward more elastic‐dominated behavior after storage, particularly for the BGP‐12 formulation. A decrease in phase angle indicates an increased contribution of elastic response relative to viscous dissipation, suggesting gradual reinforcement of reversible physical associations within the internal structure during storage. This time‐dependent increase in elasticity is consistent with pectin‐containing emulsions, where ongoing molecular rearrangement, hydration, and strengthening of protein–polysaccharide interactions can enhance the extent of reversible physical associations within the continuous phase over time [30].

Overall, the combined viscosity and phase angle results demonstrate that both conventional mayonnaise and BGP‐12 exhibit good short‐term rheological stability, with BGP‐12 showing a slightly more pronounced evolution toward elastic dominance. This behavior supports the effectiveness of the protein–polysaccharide fat‐mimetic system in maintaining structural integrity during storage while allowing controlled structural reinforcement rather than deterioration.

### 3.5. Perception of Individual Sensory Evaluation and Macronutrients Improvement

**Figure 8:**
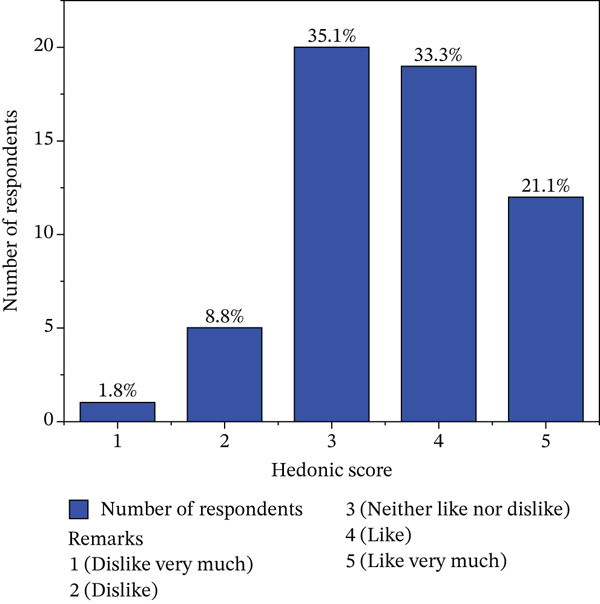
Distribution of overall liking scores of the low‐fat mayonnaise formulation based on a 5‐point hedonic scale evaluated by untrained consumers.

Table 4 presents the hedonic evaluation of individual sensory attributes for the selected low‐fat mayonnaise formulation (BGP‐12), whereas Figure 8 illustrates the distribution of overall liking scores based on a 5‐point hedonic scale. Among all evaluated attributes, viscosity achieved the highest liking score (4.11 ± 0.70), with an acceptance rate of 84.2%, indicating that the perceived thickness of the formulation closely matched consumer expectations for mayonnaise. This strong sensory response is consistent with the rheological characteristics of BGP‐12, which exhibits a relatively high storage modulus (G ^′^), a low phase angle (*δ*), and an intermediate flow behavior index (n). Together, these parameters describe a system that behaves as an elastic, structured material at rest while remaining readily deformable under shear, a combination that is widely recognized as desirable for mayonnaise‐type products during spreading and oral processing [31].

**Table 4:** Mean hedonic scores (± SD) and acceptance rates (percentage score 4–5) of individual sensory attributes of the low‐fat mayonnaise evaluated by untrained consumers using a 5‐point hedonic scale.

Attribute	Mean	SD	% acceptance (4–5)
Aroma	3.40	0.92	43.9%
Taste	3.53	1.04	63.2%
Texture	3.58	1.00	64.9%
Viscosity	4.11	0.70	84.2%
Color	3.54	0.80	59.6%

Texture and taste also demonstrated moderate to high acceptance levels, with more than 60% of respondents providing positive scores. The favorable texture perception aligns with the homogeneous microstructure observed for the BGP‐12 formulation, suggesting a smooth mouthfeel without pronounced graininess or excessive particulate perception. The positive response to taste indicates that incorporation of the protein–polysaccharide fat‐mimetic system and BGPs did not adversely affect overall palatability. Moreover, the nutty and mildly savory flavor notes associated with whey protein and black garlic were positively received by approximately 80% of respondents, indicating effective integration of these components into the flavor profile rather than the introduction of off‐notes.

In contrast, aroma received the lowest mean score (3.40 ± 0.92), which can be attributed to the distinctive volatile compounds characteristic of black garlic. Nevertheless, the score remained close to the neutral point on the hedonic scale, suggesting consumer tolerance rather than rejection. This finding indicates that aroma represents a potential area for further formulation optimization rather than a critical limitation of the system.

**Figure 9:**
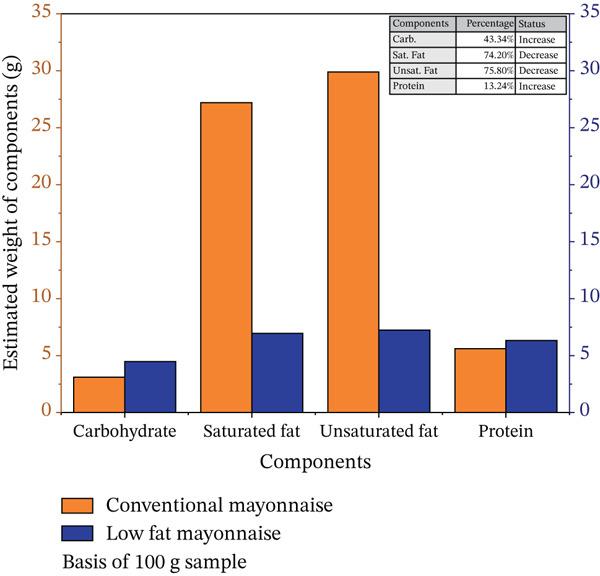
Estimated macronutrient composition of conventional and low‐fat mayonnaise formulations on a 100‐g basis.

Figure 9 summarizes the estimated macronutrient composition of the low‐fat mayonnaise in comparison with the conventional formulation on a 100‐g basis. The low‐fat formulation exhibits a pronounced reduction in both saturated and unsaturated fat contents, amounting to decreases of 74.2% and 75.8%, respectively. Accordingly, the reported fat reduction is referenced to a conventional egg yolk–based mayonnaise formulation, which typically derives lipids from both oil and egg yolk. This substantial reduction in lipid fraction is accompanied by corresponding increases in carbohydrate (43.3%) and protein (13.24%) contents, reflecting the replacement of oil with a protein–polysaccharide‐based fat‐mimetic system rather than a simple reduction in total solids.

Since lipids contribute significantly more energy (~9 kcal g^−1^) than carbohydrates or proteins (~4 kcal g^−1^), this redistribution of macronutrients leads to a marked decrease in overall caloric density. Importantly, the observed calorie reduction is not achieved through dilution with water alone, which typically compromises texture and stability, but through structural redesign of the formulation. The incorporation of WPC and pectin provides bulk, viscosity, and elastic structuring that compensates for the reduced oil volume fraction, allowing the system to maintain functional integrity despite the lower fat content.

As demonstrated by the rheological and sensory results discussed previously, this compositional shift does not adversely affect key quality attributes. The low‐fat mayonnaise retains elastic‐dominated viscoelastic behavior, appropriate shear‐thinning flow characteristics, and high sensory acceptance, particularly with respect to viscosity and texture. These findings indicate that the protein–polysaccharide fat mimetic effectively replaces not only the caloric contribution of fat but also its structural and functional roles within the emulsion.

Collectively, the macronutrient and caloric profiles highlight that meaningful reductions in both fat and energy content can be achieved while preserving the structural, rheological, and sensory characteristics expected of mayonnaise. This outcome underscores the feasibility of the proposed formulation strategy for developing reduced fat‐emulsified foods that balance nutritional improvement with consumer‐relevant quality attributes.

## 4. Conclusions

This study demonstrates that BGP‐based fat mimetics influence the physical properties and sensory acceptance of low‐fat mayonnaise. The combined use of WPC and HMP provided a structured matrix in which BGP acted as a multifunctional component, contributing to the structural organization and stability of the emulsion system. FTIR analysis indicated the presence of complementary functional groups consistent with reversible protein–polysaccharide interactions, which were reflected in the observed microstructural homogeneity and rheological behavior.

Among the tested formulations, BGP‐12 exhibited the closest resemblance to conventional mayonnaise, characterized by a consistency index of 3.87 Pa·s^n^ and shear‐thinning behavior (n = 0.45), along with enhanced viscoelastic properties (G ^′^ > G ^″^) as evidenced by a decreasing phase angle (20°–5°) with increasing angular frequency, and good short‐term stability. Furthermore, this formulation has been well received for overall sensory acceptance, particularly in viscosity (4.11 ± 0.70; 84.2%) and texture (3.58 ± 1.00; 64.9%), indicating that the perceived mouthfeel closely matched consumer expectations. Notably, the formulation achieved approximately 75% fat reduction and an estimated 45%–55% decrease in caloric content, while maintaining key quality attributes.

From an industrial perspective, these findings demonstrate the potential of BGP‐based fat mimetics as an approach for developing reduced fat emulsions with desirable structural and sensory properties. However, the study was limited to short‐term stability (7 days), and further work is needed to assess long‐term shelf life, optimize flavor and aroma, and evaluate process scalability under industrial conditions.

Overall, the results highlight the effectiveness of BGP‐based fat mimetics in structurally redesigning low‐fat mayonnaise to preserve physical functionality and sensory acceptance. This approach offers a promising strategy for developing reduced fat‐emulsified foods without compromising consumer‐relevant quality attributes.

## Funding

This study was supported by the Directorate of Research and Community Services (DPPM), Ministry of Higher Education, Science, and Technology (Kemdiktisaintek) Republic of Indonesia (496/UN.62.21/PG.00.01/2025).

## Ethics Statement

The sensory evaluation involved adult volunteers who were informed about the study and provided informed consent prior to participation. All samples were prepared and handled following strict hygiene and food safety practices.

## Conflicts of Interest

The authors declare no conflicts of interest.

## Data Availability

The data that support the findings of this study are available on request from the corresponding author. The data are not publicly available due to privacy or ethical restrictions.
